# Use of Intraoperative Frozen Section in the Surgical Management of Patients with Nonmelanoma Skin Cancer

**DOI:** 10.1155/2021/4944570

**Published:** 2021-11-01

**Authors:** LiAnn Loh, Priya Tiwari, Jingtzer Lee, O-Wern Low, Vigneswaran Nallathamby, Hanjing Lee, Jane Lim, Thiam Chye Lim, Yan Lin Yap

**Affiliations:** ^1^NUS Yong Loo Lin School of Medicine, National University of Singapore, Singapore; ^2^Division of Plastic, Reconstructive and Aesthetic Surgery, Department of Surgery, National University Hospital, Singapore; ^3^Division of Plastic, Reconstructive and Aesthetic Surgery, Department of Surgery, Ng Teng Fong Hospital, Singapore

## Abstract

**Background:**

Intraoperative frozen section (IFS) is often utilised in the surgical treatment of nonmelanocytic skin cancer (NMSC) in sensitive facial regions when Mohs micrographic surgery (MMS) is not available.

**Objective:**

To compare the outcome of NMSC patients with excision performed with and without IFS.

**Materials and Methods:**

A retrospective, single-centre study was performed on all patients who had undergone resection of NMSC with and without IFS control at the National University Hospital (NUH) from 2010 to 2015.

**Results:**

116 patients were recruited, of which 86 had IFS and 30 did not. The complete excision rate of patients with IFS was higher at 87.2% (*p*=0.0194), need for secondary operation was lower at 1.2% (*p*=0.005), and need for postsurgery radiotherapy or chemotherapy was lower at 1.2% (*p*=0.001). The average duration of surgery in patients who underwent IFS was 95.4 minutes compared to 70.1 minutes in cases which did not undergo IFS.

**Conclusion:**

Our study showed an increased complete excision rate and reduced need for secondary surgeries and adjuvant therapy in patients with IFS. However, a longer operative duration was required. Use of IFS may be useful in patients with NMSC lesions in sensitive regions requiring complex reconstruction after tumour excision.

## 1. Introduction

Nonmelanocytic skin cancer (NMSC) is one of the most common cancers in the world [1], with worldwide incidence increasing [1]. This can be attributed to factors such as increased exposure to ultraviolet light, increased outdoor activities, increased longevity, ozone depletion, and genetics [2].

Skin cancer is one of the top ten most common cancers in Singapore, and local incidence is on the rise [3, 4]. It is the 6^th^ most common cancer in males and 7^th^ most common in females [5]. NMSC, mainly basal cell carcinoma (BCC) (62%) and squamous cell carcinoma (SCC) (32%), form the majority of skin cancers [6] as compared to melanoma (6%).

The gold standard surgical treatment is Mohs micrographic surgery (MMS) or surgical excision with intraoperative histological and margin assessment [7]. Dr. Frederic Mohs first described the original technique using zinc chloride paste in the 1940s. MMS is performed under local anaesthesia with the tumor being excised at an oblique angle and horizontal sections sent for microscopic evaluation of the peripheral and deep margins. Residual tumor identified is marked on a pictorial map (Mohs map) to guide the removal in subsequent stages until negative margins are achieved [8]. MMS allows surgeons to ensure definitive excision with minimal loss of normal surrounding tissue, thereby offering high cure rates with good cosmesis [8]. It is typically indicated in patients with aggressive malignant features such as morpheaform or sclerosing subtypes, lesions near cosmetically sensitive areas (periorbital, periauricular, and paranasal), lesions with high-risk features, poorly delineated margins in scar tissue, or recurrent tumors [9]. Conventional surgical excision is used to treat small, low-risk tumours [10].

Other surgical treatment modalities and indications are as follows: electrodesiccation and curettage is best used in patients with low-risk NMSC with clear margins [11]. Cryosurgery is effective in providing high cure rates in patients with well-demarcated tumours that are not tethered to deeper structures [12]. These methods tend to leave a round scar [13] with a less-acceptable cosmetic outcome such as dyspigmentation [12, 13].

Intraoperative frozen section (IFS) of margins is performed by plastic surgeons and is seen as an optional tool which can be used as an alternative to MMS [8, 14, 15]. The National Comprehensive Cancer Network (NCCN) recommends that excision with complete circumferential peripheral and deep margin assessment is acceptable as an alternative to Mohs surgery provided it includes a complete assessment of all deep and peripheral margins [11].

IFS boasts several advantages. Surgical treatment of NMSC of the eyelids and canthi had a success rate of 87.5% with the use of IFS as compared to 69.77% without it [16]. Surgery for basal cell carcinoma including the eyelid margins with IFS and immediate plastic reconstruction was associated with better long-term cure without recurrences during a 5-year follow-up period, which was comparable to MMS [11, 16, 17]^,^. IFS was also reported to produce a cosmetically acceptable outcome in addition to a curative resection, all in one procedure that has equivalent safety and efficacy as MMS [1].

Disadvantages of IFS include false negative results as high as 19.5% [12] and 28.7% [13] due to incomplete excisions [13] and higher recurrence rates compared to MMS (3.5%) [14] and conventional excision (4.2%) [15]. A study by Nicoletti et al. demonstrated the paradoxical ineffectiveness of IFS compared to radical excision due to intrinsic technical limits of being unable to identify the margins of the NMSC [15]. Yet another drawback compared to conventional excision without IFS includes increased costs from increased operating time due to histological examination and expertise required to process and interpret the frozen section results [17].

IFS of margins is beneficial in selected groups of patients with the following features: the lesions are located in sensitive areas which may be disfiguring, patients who require reconstruction in the same setting [18, 19], and those want to avoid a subsequent reoperation for involved areas. IFS may also be useful in patients with incompletely excised lesions, lesions at sites of high risk for incomplete excision, and for recurrent lesions [20, 21]. IFS is a good alternative to MOHS when the option of MOHS surgery is unavailable. We hypothesise that this subgroup of patients will be able to enjoy a better clinical outcome which would justify increased costs and resource expenditure associated with IFS.

## 2. Aim

This retrospective study aims to review our institution's outcomes with IFS in surgical excision of NMSC.

### 2.1. Materials and Methodology

Domain-Specific Review Board (DSRB) approval was obtained from the National Healthcare Group prior to the study. A retrospective chart review was performed of all patients who underwent resection of NMSC with IFS control and those without at NUH over a 6-year period from 2010 to 2015.

Using IFS, 5 margins were taken in 1 mm strips each, 4 around the lesion labelled superior, inferior, medial, and lateral margins and 1 taken from the base. These were sent to the lab for processing and histopathological examination. If any margin was involved, additional 1 mm margins would be taken until it was reported to be clear.

No patient contact was required as data were retrieved from electronic medical records. Data collected covered the following: patient demographics include age, gender, family history, risk factors, and comorbidities. Cancer demographics include the type of cancer, location, and sublocation of the cancer, tumour size, and tumour stage. Operative details only excisional surgeries included in study, duration of surgery, whether an intraoperative frozen section was carried out, frozen section results, and final histological results. Hospitalization data include length of inpatient stay and complications. Outcome data include recurrence and the need for secondary operation or adjuvant treatment.

The data were processed using the Statistical Package for Social Sciences (SPSS) software. The frequency, percentage, and mean were calculated for each variable. The chi-square test was used to analyse the associations between patient demographics, cancer demographics, and operative details. A *p* value of <0.05 indicated the result was statistically significant.

## 3. Results

### 3.1. Patient and Cancer Demographics

A total of 116 cases were included in this retrospective study. The mean age of all patients was 71.6 years (range: 24–100 years) at the time of operation. There was an even distribution of males (50%) and females (50%) selected. 74.1% (*n* = 86) of all patients had IFS performed while 25.9% (*n* = 30) did not (Table 1).

At the time of surgery, the mean age of patients in the group where IFS was performed was 72.8 years while the mean age of patients in the group without IFS performed was 68.3 years. In both groups, the head and neck region was most frequently affected (72.4%, *n* = 84).

The diagnosis of the NMSC was ascertained in some patients, for example, those who had tumours near vital structures prior to excision and reconstructive surgery. 42.2% of all patients (*n* = 49) had preoperative biopsy performed, while the remaining 57.8%(*n* = 67) did not. BCCs accounted for 66.4% (*n* = 77) of all lesions, followed by SCCs at 21.6% (*n* = 25). The remaining 12% (*n* = 14) of lesions include Bowen's disease, keratoacanthoma, pigmented nevus, porocarcinoma, and dermatofibrosarcoma protuberans. There was no statistically significant difference in the size of tumour between these 2 groups of patients.

### 3.2. Intraoperative and Postoperative Outcomes

Margins were clear in 87.2% (*n* = 75) of cases with IFS compared to 67.9% (*n* = 19) in those without (*p*=0.0194). Pathology specimens are embedded en-face to ensure complete histological evaluation of specimens. The intraoperative duration, defined as time taken from skin incision to completion of surgery including reconstruction, took over 90 minutes in 41.9% (*n* = 36) of cases with IFS compared to 33.3% (*n* = 10) in those without (*p*=0.0192). The average duration for patients who underwent IFS was 95.4 mins and 70.1 mins for those without IFS. 88.4% (*n* = 76) of cases with IFS had more complex reconstruction, namely, skin grafting and locoregional flaps, performed in comparison to 53.3% (*n* = 16) of cases without IFS (Table 2).

The mean duration of follow-up was 73.7 months, with a minimum follow-up period of 5 years for all patients. With IFS use, only 1.2% (*n* = 1) recurred, compared to 6.67% (*n* = 2) in the group without IFS. In the group of patients with IFS, 1.2% (*n* = 1) required a second surgery and 1.2% (*n* = 1) required further treatment. The remaining 9 patients with involved surgical margins were offered options of reexcision, and only 1 patient agreed and underwent a reexcision of margins and defect coverage with full-thickness skin graft. For the other 8 patient with involved surgical margins, 3 were lost to follow-up and 5 remain on dermatology skin cancer surveillance follow-up and have undergone cryotherapy for actinic keratosis. This is in comparison to the group without IFS, where 13.3% (*n* = 4) required a second surgery and 16.7% (*n* = 5) required further treatment. Of these results, the reduction in need for a second operation and further treatment in the group with IFS compared to the group without IFS were statistically significant, where *p*=0.005 and *p*=0.001, respectively.

## 4. Discussion

The majority of NMSCs operated on in our institution occur in the head and neck region. This is a particularly sensitive area because of the need to obtain cancer-free margins and ensure the best cosmetic and functional outcomes. IFS of margins offers surgeons the benefit of ascertaining clear margins while keeping the size of resection and, hence, degree of reconstruction to a minimum.

In contrast to other studies, our review yielded a small false negative rate of 1.33%. This is lower than the 2.47%–27.8% false negative rate reported in other studies on the use of IFS [13, 14, 22]. This is due to the availability of multidisciplinary on-site dermopathology expertise.

Patients with IFS performed showed a complete excision rate that is statistically significant. Both groups did not show a difference in recurrence rate. However, the reduction in need for a second operation and further treatment in the group with IFS performed was statistically significant. These findings prove that the use of IFS confers benefits to patients. While the financial cost of an IFS procedure is higher, this is offset by a lower emotional and mental burden due to the lower probability of requiring a second surgery or other treatment modalities such as radiotherapy and chemotherapy.

With IFS, surgeons are able to proceed with more complicated reconstruction at the primary resection surgery, namely, skin grafting and use of locoregional flaps, with the confidence of ensuring clear margins intraoperatively in such patients.

However, there is a tradeoff when employing IFS. In the group with IFS performed, a statistically significant number took longer for the surgery to be completed, due to waiting for intraoperative frozen section results from the pathologist, compared to the group without IFS. The longer operative durations may put patients at risk of further postoperative complications [16] from the surgery itself and the prolonged exposure to anaesthetic agents. Another point against the use of IFS is the additional cost [16]. In our institution, patients would be charged an additional SGD$1,500 to $2500 approximately for intraoperative frozen sections.

There are several limitations seen in our retrospective study.

Firstly, our study had a small sample size of only 116 cases. Of these, we note that there was an uneven division such that there were 56 more patients in the group that had IFS performed. This disproportion could have skewed the results of the study.

Next, an extended operating time was seen in 41.9% of cases in the group which had IFS performed as compared to 33.3% in the group without it, and there may have been other intraoperative factors contributing to this longer duration, such as the lesions being closer to vital structures, thus making surgeries more challenging and surgeries being performed by different surgeons with diverse experience.

Lastly, the exact cost of surgery with and without the inclusion of IFS was not collected in the data. As such, comparison of the cost difference was not able to be performed. In order to analyse the costs and benefits of this procedure more accurately, it would be useful to collate this particular dataset.

## 5. Conclusions

Although the use of IFS currently remains a controversial topic [19], our retrospective study has demonstrated that its use in our institution has yielded a higher rate of complete excisions with low false negative results. Despite the drawbacks of an extended operating time and increased surgical costs, patients can still enjoy a multitude of benefits where the balance between complete excision and best cosmetic outcome is achieved. Patients with lesions in the head and neck region would require greater preservation of both form and function and, thus, would most benefit from IFS analysis in the treatment of NMSC. Another group of patients who would greatly benefit from the use of IFS in NMSC treatment includes those requiring more complex reconstruction after tumour excision, as surgeons have increased confidence of clear margins prior to performing complex reconstructions.

## Figures and Tables

**Table 1:** Patient and cancer demographics.

	IFS performed (*N* = 86)	IFS not performed (*N* = 30)	*p* value
Mean age at the time of surgery	72.8 years (range: 24–95)	68.3 years (range: 24–100)	
Gender			
Male	45	13	0.396
Female	41	17	
Tumour location			
Head and neck	61	23	0.513
Trunk	4	2	
Upper limb	15	2	
Lower limb	6	3	
Biopsy performed			
Punch/shave	23	8	0.465
Incision	9	1	
Excision	7	1	
Tumour size			
<1 × 1 cm	20	10	0.447
>1 × 1 cm	32	7	
>2 × 2 cm	29	12	
Missing data	5	1	
Preoperative diagnosis			
BCC	57	20	0.185
SCC	21	4	
Others	8	6	

**Table 2:** Intraoperative and postoperative outcomes.

	IFS performed (*N* = 86)	IFS not performed (*N* = 30)	*p* value
Margins			
Clear	75	19	0.0194
Not clear	11	9	
Intraoperative duration			
Duration >90 min	36	10	0.0192
Duration <90 min	50	20	
Type of reconstruction			
Primary closure	9	14	0.0003
Skin grafting	45	11	
Locoregional flap	31	5	
VAC dressing	1	0	
Tumour recurrence			
Yes	1	2	0.102
No	85	28	
Second operation			
Yes	1	4	0.005
No	85	26	
Further treatment			
Yes	1	5	0.001
No	85	25	

## Data Availability

The Excel file with patient demographics and skin cancer type, excision margins, and histology data used to support the findings of this study is available from the corresponding author upon request.

## References

[1] Lomas A., Leonardi‐Bee J., Bath‐Hextall F. (2012). A systematic review of worldwide incidence of nonmelanoma skin cancer. *British Journal of Dermatology*.

[2] Leiter U., Eigentler T., Garbe C. (2014). Chapter 7: Epidemiology of skin cancer. *Sunlight, Vitamin D and Skin Cancer*.

[3] Kim G. K., Del Rosso J. Q., Bellew S. (2009). Skin cancer in asians Part 1: nonmelanoma skin cancer. *The Journal of Clinical and Aesthetic Dermatology*.

[4] Koh D., Wang H., Lee J., Chia K. S., Lee H. P., Goh C. L. (2003). Basal cell carcinoma, squamous cell carcinoma and melanoma of the skin: analysis of the Singapore Cancer Registry data 1968-97. *British Journal of Dermatology*.

[5] (2016). Common types of cancer. https://www.singaporecancersociety.org.sg/learn-about-cancer/cancer-basics/common-types-of-cancer-in-singapore.html.

[6] Sng J., Koh D., Siong W. C., Choo T. B. (2009). Skin cancer trends among Asians living in Singapore from 1968 to 2006. *Journal of the American Academy of Dermatology*.

[7] Attia A. B. E., Chuah S. Y., Razansky D. (2017). Noninvasive real-time characterization of non-melanoma skin cancers with handheld optoacoustic probes. https://www.sciencedirect.com/science/article/pii/S2213597917300113.

[8] Cumberland L., Dana A., Liegeois N. (2009). Mohs micrographic surgery for the management of nonmelanoma skin cancers. *Facial Plastic Surgery Clinics of North America*.

[9] Nehal K. S., Bichakjian C. K. (2018). Update on keratinocyte carcinomas. *New England Journal of Medicine*.

[10] Amjadi M., Coventry B., Greenwood J. (2011). Surgical treatments of non-melnaoma skin cancers: a review. *The Internet Journal of Plastic Surgery*.

[11] (2020). Nonmelanoma skin cancer (NMSC): signs, symptoms and treatments. https://skinofcolorsociety.org/dermatology-education/nonmelanoma-skin-cancer-nmsc/.

[12] Holt P. J. A. (1988). Cryotherapy for skin cancer: results over a 5-year period using liquid nitrogen spray cryosurgery. *British Journal of Dermatology*.

[13] Wang X. L., Wang B., Shi L. (2017). Gain with no pain? Pain management in dermatologic photodynamic therapy. *British Journal of Dermatology*.

[14] Moncrieff M. D., Shah A. K., Igali L., Garioch J. J. (2015). False-negative rate of intraoperative frozen section margin analysis for complex head and neck nonmelanoma skin cancer excisions. *Clinical and Experimental Dermatology*.

[15] Chambers K. J., Kraft S., Emerick K. (2014). Evaluation of frozen section margins in high-risk cutaneous squamous cell carcinomas of the head and neck. *The Laryngoscope*.

[16] Nicoletti G., Brenta F., Malovini A., Musumarra G., Scevola S., Faga A. (2012). Study to determine whether intraoperative frozen section biopsy improves surgical treatment of non-melanoma skin cancer. *Molecular and Clinical Oncology*.

[17] Chren M.-M., Torres J. S., Stuart S. E., Bertenthal D., Labrador R. J., Boscardin W. J. (2011). Recurrence after treatment of nonmelanoma skin cancer. *Archives of Dermatology*.

[18] Nizamoglu M., Douglas H., Mcardle C., Mathew B., Vize C., Matteucci P. (2016). Using frozen section margin control technique to manage non-melanomatous skin lesions in high-risk sites. *Journal of Plastic, Reconstructive & Aesthetic Surgery*.

[19] Benedict K. C., Bilden T. T., Lamb P., Mcmullin J. (2019). Intraoperative frozen section analysis for the excision of nonmelanoma skin cancer. *The American Surgeon*.

[20] Fogarty B. J., Khan K., Ashall G., Leonard A. G. (1999). Complications of long operations: a prospective study of morbidity associated with prolonged operative time (>6 h). *British Journal of Plastic Surgery*.

[21] Dinardo L. J., Lin J., Karageorge L. S., Powers C. N. (2000). Accuracy, utility, and cost of frozen section margins in head and neck cancer surgery. *The Laryngoscope*.

[22] Conway R. M., Themel S., Holbach L. M. (2004). Surgery for primary basal cell carcinoma including the eyelid margins with intraoperative frozen section control: comparative interventional study with a minimum clinical follow up of 5 years. *British Journal of Ophthalmology*.

